# Supplementary material for: The Structure Properties of Carbon Materials Formed in 2,4,6-Triamino-1,3,5-Trinitrobenzene Detonation: A Theoretical Insight for Nucleation of Diamond-like Carbon
Source: Int J Mol Sci. 2023 Aug 8;24(16):12568. doi: 10.3390/ijms241612568 (PMC10454052; doi:10.3390/ijms241612568)
Supplement: Supplementary file 1 [file ijms-24-12568-s001.zip › ijms-2502440-SI.pdf]

Article

The Structure Properties of Carbon Materials Formed in 2,4,6-Triamino-1,3,5-Trinitrobenzene Detonation: A Theoretical Insight for Nucleation of Diamond-like Carbon

Zheng-Hua He [1], Yao-Yao Huang [1], Guang-Fu Ji [1], Jun Chen [2,*] and Qiang Wu [1,*]

[1] National Key Laboratory of Shock Wave and Detonation Physics, Institute of Fluid Physics, China Academy of Engineering Physics, Mianyang 621900, China; herary-hezhh@caep.cn (Z.-H.H.); huangyaoyao18@gscaep.ac.cn (Y.-Y.H.); cyfjkf@126.com (G.-F.J.)
[2] National Key Laboratory of Computational Physics, Institute of Applied Physics and Computational Mathematics, Beijing 100088, China
* Correspondence: jun_chen@iapcm.ac.cn (J.C.); wuqiang@caep.cn (Q.W.)

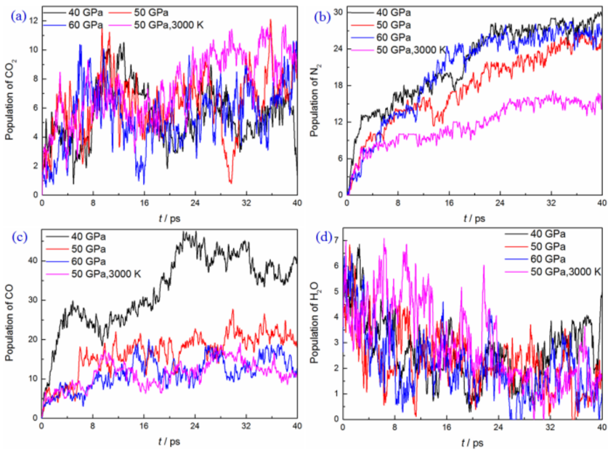

Figure S1. Population evolutions of main species involved in carbon cluster deformation at first 40 ps.

The component and evolution of early gaseous products (CO, CO_2, N_2, and H_2O) are investigated and shown in Fig. S1. As reaction progress, many CO and N_2 molecules are produced, and their populations gradually increase to reach their maxima. In comparison, only few CO_2 and H_2O molecules are formed, and their populations drastically fluctuate during reaction process. It indicates that they possess high reaction activities under such high temperature and pressure. Besides, low

pressure can significantly promote the formation of CO molecule, while high temperature is beneficial to form N_2 molecule. However, no obvious dependences on the temperature and pressure are observed about the generation of CO_2 and H_2O.

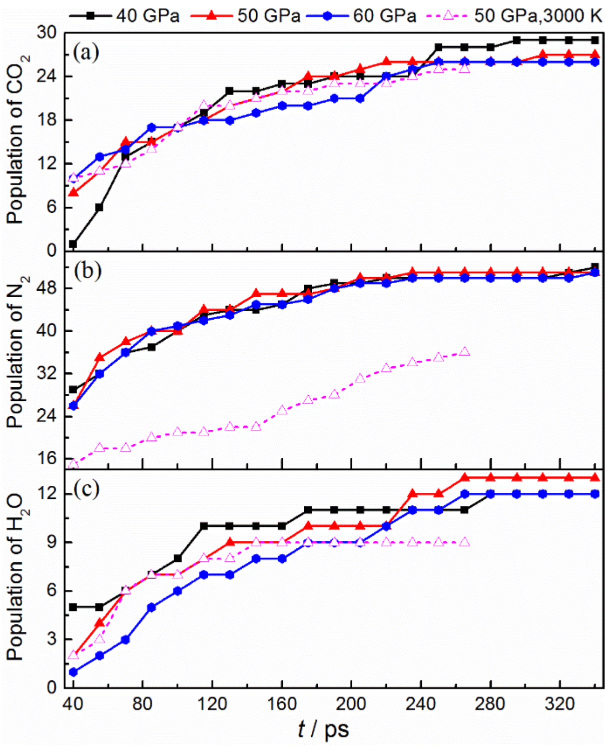

Figure S2. Population evolutions of main stable gaseous molecules (the solid lines represent the conditions of 3500 K; dot line represents condition of 3000 K).

The stable molecules of CO_2, N_2, and H_2O are separated from reaction system every 15 ps, and their accumulative numbers are shown in Fig. S2. At 3500 K (solid lines), Low pressure is obvious beneficial for the C and H oxidation to form more CO_2 and H_2O molecules, but no similar effect on the formation of N_2 is observed. On the contrary, high temperature can efficiently promote the N atom release, with more N_2 molecule generation (see Fig. S2b), while no significant impacts of temperature on CO_2 and H_2O formation are detected.

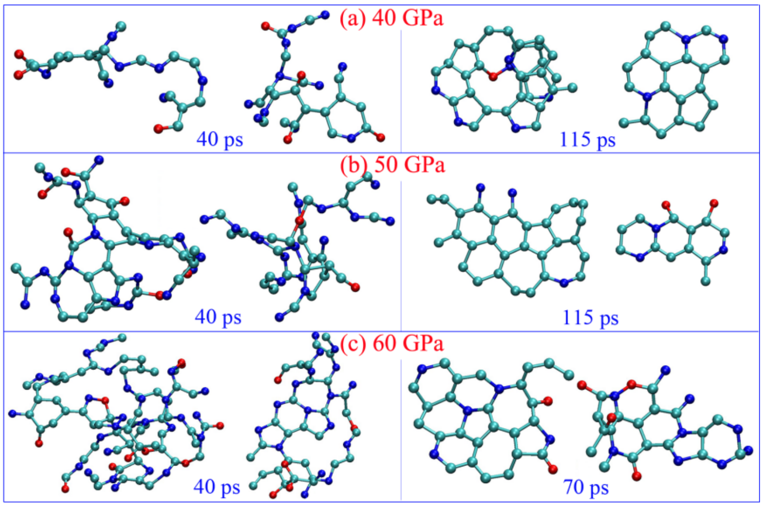

Figure S3. Microscopic structure of the main fragments involved in carbon cluster evolution process with different pressures.

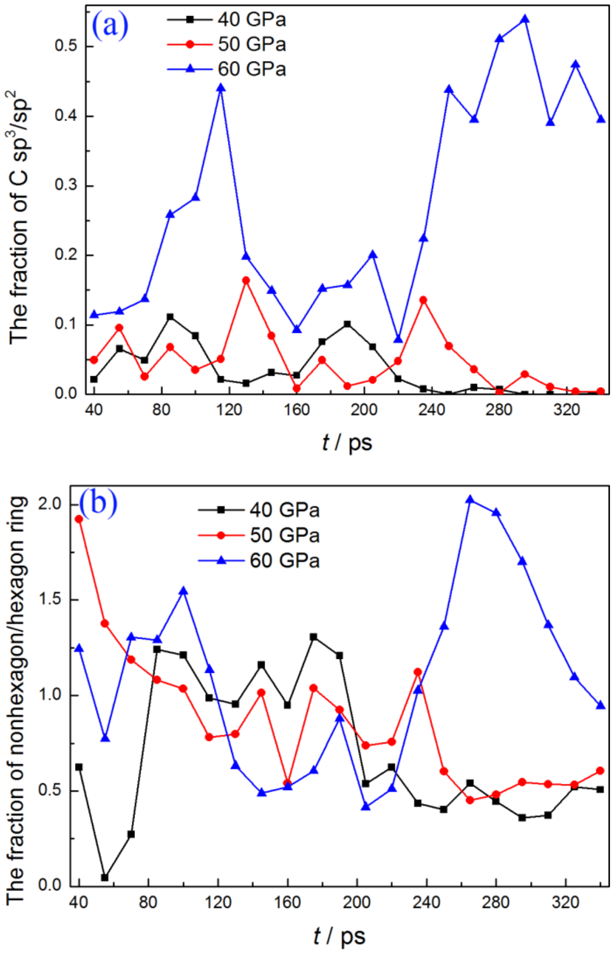

Figure S4. The fraction of C sp^3 to sp^2 (a) and nonhexagon to hexagon ring (b) under different

pressures.

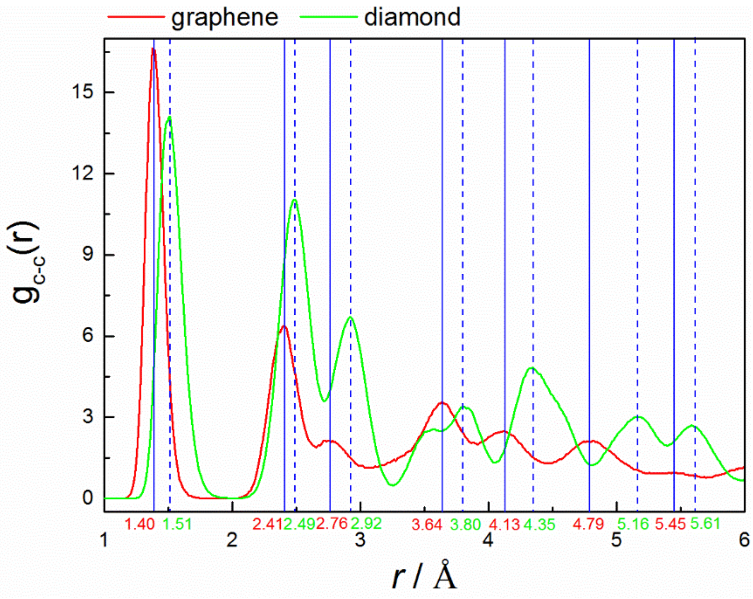

Figure S5. Radial pair distribution functions of g_{c-c} for the graphene and diamond under 50 and 60 GPa, respectively.

Stable gaseous products remove strategy: The stable molecules components are confirmed using the bond-length and lifetime criteria, which define the critical interaction distance and time. Any atom pairs satisfying above criteria are considered to be bonded, and any atoms bonding with each other belong to the same molecule. The initial carbon cluster is relaxed at first 40 ps, and the CO_2, N_2, and H_2O molecules are separated from reaction system. After that, these stable gaseous molecules are gradually eliminated every 15 ps to accelerate the purification and coagulation of carbon cluster.
